# Partial pericardiectomy for refractory acute tuberculous pericarditis: A case report

**DOI:** 10.1016/j.ijscr.2023.108239

**Published:** 2023-04-19

**Authors:** André Loureiro Fernandes, Fabrício José Dinato, Elinthon Tavares Veronese, Carlos Manuel de Almeida Brandão, Vera Demarchi Aiello, Fabio Biscegli Jatene

**Affiliations:** a Division of Cardiovascular Surgery, Heart Institute, University of São Paulo Medical School, São Paulo, Brazil; b Department of Pathology, Heart Institute, University of São Paulo Medical School, São Paulo, Brazil

**Keywords:** Pericarditis, Tuberculous pericarditis, *Mycobacterium tuberculosis*, Pericardiectomy

## Abstract

**Introduction:**

Tuberculosis is an infectious disease that usually manifests in the lungs but can also affect other organs, including the cardiovascular system. In this article, we present a rare case of purulent pericarditis caused by *Mycobacterium tuberculosis.*

**Presentation of case:**

A 67-year-old man was admitted to the emergency department with a large pericardial effusion with evidence of cardiac tamponade caused by acute pericarditis. The patient underwent surgical pericardial drainage, and a total volume of 500 mL of purulent fluid was collected with a positive culture for *Mycobacterium tuberculosis*. Despite antituberculous drugs, the patient presented with clinical worsening and recurrence of large pericardial effusion. Therefore, he was submitted to a second intervention by full median sternotomy to drain the pericardial effusion and perform a surgical pericardial debridement associated with a partial pericardiectomy. After the procedure, he improved clinically and was discharged after 24 days of hospitalization.

**Discussion:**

Pericardiectomy is recommended for patients with refractory tuberculous pericarditis after four to eight weeks of antituberculous treatment. We decided not to wait that long to perform an open surgical partial pericardiectomy and debridement with a median sternotomy approach. We believe that this more aggressive surgical approach would be more efficient to combat the infection, which was causing progressive deterioration of patient's clinical condition and early recurrence of significant pericardial effusion.

**Conclusion:**

Open partial pericardiectomy with surgical debridement could be an efficient approach for treatment of a refractory acute tuberculous pericarditis.

## Highlights

• Pericardial disease rarely occurs as the initial manifestation of tuberculosis.
• Tuberculous pericarditis should be in the differential diagnosis of purulent pericarditis.
• Tuberculous pericarditis can manifest without other signs of active tuberculosis.
• In case of unsatisfactory response to clinical treatment, partial pericardiectomy can be effective in controlling tuberculous pericarditis.

## 1 Introduction

Tuberculosis is an infectious disease caused by *Mycobacterium tuberculosis,* an acid-alcohol-resistant bacilli. It is transmitted by inhalation of aerosols exhaled by a person with active pulmonary or laryngeal tuberculosis. It is estimated that only 10 % of persons infected with *Mycobacterium tuberculosis* will develop a clinical manifestation of the disease [1]. Pulmonary tuberculosis is the most common clinical manifestation; however, other systems such as the nervous, digestive and cardiovascular systems may also be affected.

When the disease presents in a cardiovascular form, pericarditis is the most important manifestation, occurring in approximately 5 % of immunocompetent patients, followed by myocarditis and aortitis [2]. Purulent pericarditis is a rare manifestation of pericarditis, accounting for <1 % of cases. It is an acute and fulminant disease that requires rapid treatment given the high mortality rate [3]. Only 3 % of pericardial effusions caused by *Mycobacterium tuberculosis* manifest as purulent pericarditis [4]. In this article, we present a rare case of purulent pericarditis caused by *Mycobacterium tuberculosis* in which a surgical partial pericardiectomy was performed as part of the treatment. This case is reported in line with SCARE criteria [5].

## 2 Presentation of case

A 67-year-old man with a history of alcoholism and former smoker was admitted to the emergency department with acute, intense, and oppressive chest pain that had begun four days earlier and was associated with exertional dyspnea. Symptoms worsen with recumbency. He denied fever, cough, and weight loss. Physical examination revealed muffled heart sounds and jugular vein distention.

**Fig. 1:**
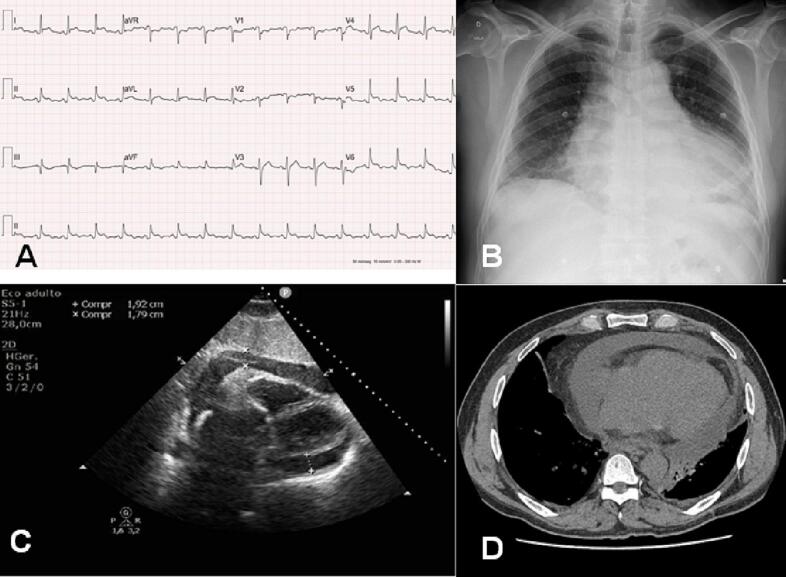
A: Electrocardiogram upon admission demonstrating diffuse ST-segment elevation and PR depression, suggesting acute pericarditis. B: Chest X-ray upon admission showing enlargement of the cardiac silhouette. C: Transthoracic echocardiogram showing pericardial effusion. D: Computed tomography revealing pericardial effusion.

Electrocardiogram (Fig. 1A) showed diffuse ST-segment elevation and PR depression. Chest radiography (Fig. 1B) showed enlargement of the cardiac silhouette. Transthoracic echocardiogram (Fig. 1C) demonstrated large pericardial effusion with evidence of cardiac tamponade and chest computed tomography (Fig. 1D) revealed pericardial effusion without evidence of mediastinal lymphadenopathy or pulmonary disease.

The patient underwent an emergency pericardial drainage with an open surgical subxiphoid approach. The total drained volume was 500 mL of purulent fluid, and pericardial thickening was noted. Biochemical analysis of the pericardial fluid revealed a pH of <6.3, lactate dehydrogenase 15,106 U/L, glucose 199 mg/dL (55 % of serum glucose), and adenosine deaminase (ADA) 160 U/L. Cytologic evaluation revealed polymorphonuclear dominance. *Mycobacterium tuberculosis* was detected in pericardial effusion's culture. Therefore, the diagnosis of tuberculous acute pericarditis was made and treatment with rifampim, isoniazid, pyrazinamide, and ethambutol was initiated. The HIV serological blood test was negative.

**Fig. 2:**
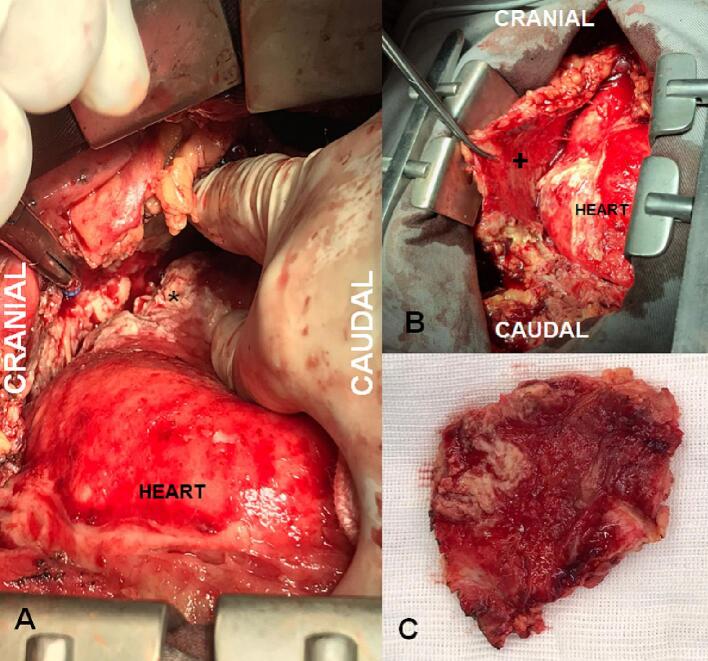
A: Surgical aspect of fibrinopurulent (*) tuberculous pericarditis. B: Purulent adhesions and thickened pericardium (+). C: Fragment of pericardium sent to histopathological analysis.

Eight days after surgical pericardial drainage, the patient developed a new large pericardial effusion visualized on transthoracic echocardiogram, associated with clinical and laboratory deterioration and paroxysmal atrial fibrillation episodes. Therefore, a second open surgical intervention was indicated, this time by full median sternotomy to drain the pericardial effusion and perform a surgical cleaning of the pericardial cavity. During the procedure, the aspect of fibrinopurulent pericarditis associated with pericardial thickening was evident. Thus, a partial pericardiectomy was performed from the right phrenic nerve to the left phrenic nerve, combined with surgical cleaning and debridement of the purulent adhesions (Fig. 2).

**Fig. 3:**
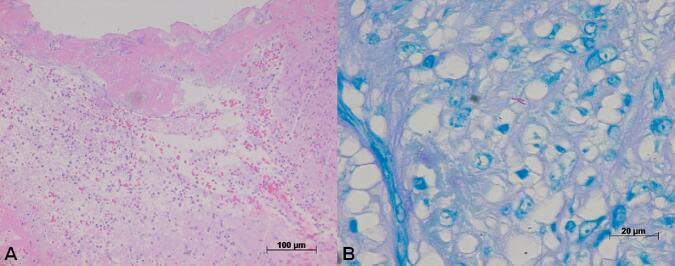
A: Photomicrograph of the pericardial surface showing exudative inflammatory infiltration with fibrin deposition. No well-formed granulomas were observed. Hematoxylin-eosin staining, objective magnification = 20×. B: Photomicrograph of a histological pericardial section stained with the Ziehl-Neelsen method, showing two acid-fast positive bacilli (in purple). Objective magnification = 100×. (For interpretation of the references to colour in this figure legend, the reader is referred to the web version of this article.)

The pericardial histological examination showed acute, diffuse an exsudative pericarditis, with a large amount of neutrophils and fibrin deposits at the surface (Fig. 3A). There were also areas of chronic inflammation, but no granulomatous reaction was observed. The Ziehl-Neelsen staining demonstrated acid-fast positive bacilli (Fig. 3B).

After open surgical pericardiectomy, the patient improved clinically, and he was discharged after 24 days of hospitalization. He continues to receive treatment to date, with a 8-month course of tuberculous pericarditis planned, without recurrence of symptoms.

## 3 Discussion

Acute pericarditis is a common manifestation of pericardial disease and accounts for up to 5 % of patients admitted to the emergency department with chest pain not due to myocardial infarction. The most prevalent etiologies are viral and idiopathic; however, several conditions can cause an acute pericardial inflammation such as autoimmune diseases, uremia, and bacterial infections [6]. Some common pathogens include Staphylococcus, Streptococcus, Haemophilis and Mycobaterium tuberculosis [3].

Despite the fact that effective treatments exist, tuberculosis remains a public health problem, especially in developing countries. In 2017, there were an estimated 10 million new cases and 1.6 million deaths from tuberculosis [4]. Pericardial involvement caused by *Mycobacterium tuberculosis*, as occurred in our patient, is rare in immunocompetent patients, happening in only 5 % of extrapulmonary tuberculosis [2].

Dissemination of bacilli to the pericardium can occur by several routes. In immunocompetent individuals, pericardial involvement usually occurs by lymphatic dissemination via paratracheal, peribronchial, and mediastinal lymph nodes or by direct dissemination from the lungs and pleura. In our patient, it was not clear how the bacilli spread to the pericardium because he had no signs of lymphadenopathy or pulmonary disease on imaging studies [2].

Etiologic diagnosis of pericarditis is usually challenging. In 15 to 20 % of cases, the causative factor is not found. The initial examination consists of a detailed clinical history and complementary exams. A chest radiograph may show evidence of concurrent pulmonary tuberculosis in 30 % of patients. Computed tomography or magnetic resonance imaging may be useful if there is thickening of the pericardium (> 3 mm) or effusion and lymphadenopathy (> 10 mm). Other tests include sputum or gastric aspirate cultures. Tuberculin Skin Test is not usually useful as a diagnostic tool [7].

Diagnostic pericardiocentesis should be considered in all patients with suspected tuberculous etiology. For definitive diagnosis, the bacilli must be isolated in culture or by direct visualization in pericardial fluid or in fragments of pericardial tissue. Although these methods have high specificity, their sensitivity is about 70 % [8].

When culture or histologic examination are inconclusive, laboratory tests may be helpful in diagnosing tuberculous pericarditis. Adenosine deaminase (ADA) is found in large amounts (> 40 U/L) in the pericardial fluid of these patients with a specificity and sensitivity of 87 % and 89 %, respectively [9]. Another biomarker is interferon-gamma (IFN- γ). It has higher sensitivity and specificity than ADA; however, its higher cost is still a barrier to its wide availability in endemic areas [10]. The Light's criteria may be useful in the study of pericardial effusion because it distinguishes between exudative and transudative fluid. In our patient, biochemical analysis suggested an exudative fluid, indicating an inflammatory cause.

In our case, the initial history and complementary imaging exams were not helpful in determining the etiology. There were no signs of active tuberculosis and imaging studies revealed no associated pulmonary disease. The diagnosis was made by *Mycobacterium tuberculosis* isolation in a pericardial fluid culture and by direct visualization in histologic examination of pericardial biopsy. Elevated adenosine deaminase in pericardial fluid and biochemical analysis were also helpful in confirming diagnosis.

The goals of treatment of tuberculous pericarditis are to control *Mycobacterium tuberculosis* infection, attenuate cardiac compression and its hemodynamic effects, and prevent the development of constrictive pericarditis [10].

Clinical therapy consists of antituberculous drugs and corticosteroids. A four-drug regimen is recommended as initial therapy (rifampim, isoniazid, pyrazinamide, and ethambutol) with a duration of two months, followed by rifampim and isonizaid for six months. Studies have shown that corticosteroid therapy reduces mortality, progression to constrictive pericarditis, and hospitalization. The usual drug is prednisone for eight weeks [2].

Pericardial drainage is indicated in patients with cardiac tamponade. Constrictive pericarditis is the most important complication of tuberculous pericarditis and occurs in 30 % to 60 % of cases. Pericardiectomy is recommended if the patient does not respond satisfactorily after four to eight weeks of antituberculous treatment [11]. We decided not to wait that long and to perform an open surgical partial pericardiectomy and debridement with a median sternotomy approach. We strongly believe that this more aggressive surgical approach would be more efficient to combat the infection, which was causing progressive deterioration of patient's clinical condition and recurrence of significant pericardial effusion in only seven days after the initial surgery [3]. Besides, due to the high degree of infectious and inflammatory involvement of pericardium, we decided to perform a partial pericardiectomy also to prevent a highly possible development of constrictive pericarditis.

It is noteworthy that due to our patient did not present any clinical signs or evidence on complementary diagnostic modalities of a constrictive component of his pericardial disease, the clinical entity of effusive constrictive pericarditis was not carefully investigated. Effusive–constrictive pericarditis is an uncommon pericardial syndrome best characterized in patients with tamponade who continue to have elevated intracardiac pressure after the removal of pericardial fluid [12]. Throughout its clinical evolution, the patient manifested more prominently with pericardial effusion and sepsis and such pericardial thickening was better identified in the pericardiectomy intraoperative period and it didn't seem to be a degree of thickening capable of causing a constrictive cardiac effect at that stage of the pericardial tuberculous disease. In any case, it is very likely that the constrictive component, if not yet present, would manifest itself in a near future if the treatment with pericardiectomy had not been performed.

After surgical debridement and pericardiectomy, the patient's clinical condition and laboratory test results improved without recurrence of pericardial fluid accumulation.

## 4 Conclusion

In conclusion, it is possible to say that an open partial pericardiectomy with surgical debridement could be an efficient approach for treatment of a refractory acute tuberculous pericarditis and can be considered in the therapeutic arsenal of this condition.

## Informed consent

Written informed consent was obtained from the patient for publication of this case report and accompanying images. A copy of the written consent is available for review by the Editor-in-Chief of this journal on request.

## Provenance and peer review

Not commissioned, externally reviewed.

## Ethical approval

Ethical approval has been exempted by our institution as this publication is a case report, provided that the patient gave her written consent both for operation and the publication of this case

## Funding

No source of funding

## Author contribution

André Loureiro Fernandes: writing and editing

Fabrício José Dinato: writing and editing

Elinthon Tavares Veronese: reviewing

Carlos Manuel de Almeida Brandão: reviewing

Vera Demarchi Aiello: writing and acquisition of data

Fabio Biscegli Jatene: critical revision

## Guarantor

Fabrício José Dinato

## Conflict of interest statement

No conflicts of interest.

## References

[1] Natarajan A., Beena P.M., Devnikar A.V., Mali S. (2020). A systemic review on tuberculosis. Indian J. Tuberc..

[2] López-López J.P., Posada-Martínez E.L., Saldarriaga C., Wyss F., Ponte-Negretti C.I., Alexander B. (2021). Neglected tropical diseases, other infectious diseases affecting the heart (the NET-Heart Project). Tuberculosis and the heart. J Am Heart Assoc..

[3] Pankuweit S., Ristić A.D., Seferović P.M., Maisch B. (2005). Bacterial pericarditis: diagnosis and management. Am. J. Cardiovasc. Drugs.

[4] Johari M.I., Ramli A.W., Mat Lawi F., Bin Fouzi M.A.H., Suardi K.P.S. (2019). A rare case of purulent pericardial TB. Cureus.

[5] Agha R.A., Franchi T., Sohrabi C., Mathew G., for the SCARE Group (2020). The SCARE 2020 guideline: updating consensus Surgical CAse REport (SCARE) guidelines. International Journal of Surgery.

[6] Snyder M.J., Bepko J., White M. (2014). Acute pericarditis: diagnosis and management. Am. Fam. Physician.

[7] Adler Y., Charron P., Imazio M., Badano L., Barón-Esquivias G., Bogaert J., Brucato A. (2015). ESC Scientific Document Group. 2015 ESC Guidelines for the diagnosis and management of pericardial diseases: the Task Force for the Diagnosis and Management of Pericardial Diseases of the European Society of Cardiology (ESC) endorsed by: the European Association for Cardio-Thoracic Surgery (EACTS). Eur Heart J..

[8] Ntsekhe M., Mayosi B.M. (2013). Tuberculous pericarditis with and without HIV. Heart Fail. Rev..

[9] Reuter H., Burgess L., van Vuuren W., Doubell A. (2006). Diagnosing tuberculous pericarditis. QJM.

[10] Isiguzo G., Du Bruyn E., Howlett P., Ntsekhe M. (2020). Diagnosis and management of tuberculous pericarditis: what is new?. Curr. Cardiol. Rep..

[11] Chang S.A. (2017). Tuberculous and infectious pericarditis. Cardiol. Clin..

[12] Syed F.F., Ntsekhe M., Mayosi B.M., Oh J.K. (2013 May). Effusive-constrictive pericarditis. Heart Fail. Rev..

